# Constitutive expression of Atlantic salmon Mx1 protein in CHSE-214 cells confers resistance to Infectious Salmon Anaemia virus

**DOI:** 10.1186/1743-422X-2-75

**Published:** 2005-08-26

**Authors:** Molly JT Kibenge, Khalid Munir, Frederick SB Kibenge

**Affiliations:** 1 Department of Pathology and Microbiology, Atlantic Veterinary College, University of Prince Edward Island, 550 University Avenue, Charlottetown, PE. C1A 4P3. Canada

## Abstract

Infectious salmon anaemia (ISA) is a highly fatal viral disease affecting marine-farmed Atlantic salmon which is caused by ISA virus (ISAV), a fish orthomyxovirus that has recently been assigned to the new genus *Isavirus *within the family *Orthomyxoviridae*. Mx proteins are among the interferon (IFN)-induced proteins responsible for the development of an antiviral state in vertebrate cells. We used real-time reverse transcription-polymerase chain reaction (RT-PCR) and Chinook salmon embryo (CHSE-214) cells constitutively expressing Atlantic salmon Mx1 protein (ASMx1) to examine the antiviral properties of ASMx1 against two ISAV strains, NBISA01 and HKS-36, having phenotypically different growth properties (cytopathic vs non-cytopathic) in the CHSE-214 cell line. We present evidence that ISAV is sensitive to ASMx1. CHSE-214 cells constitutively expressing ASMx1 showed increased resistance to infection with the cytopathic ISAV strain NBISA01, manifested as delayed development of cytopathic effects (CPE) and significant reduction in the severity of CPE, as well as a 10-fold reduction in virus yield. However, by real-time RT-PCR we observed no significant difference in the mean threshold cycle (Ct) values of ISAV RNA levels, suggesting that the ASMx1 activity on ISAV occurs at the post-transcription steps of virus replication, possibly in the cytoplasm.

## Findings

Infectious salmon anaemia (ISA) virus (ISAV), the causative agent of a highly fatal disease of marine-farmed Atlantic salmon, is a fish orthomyxovirus that has recently been assigned to the new genus *Isavirus *within the family *Orthomyxoviridae *[1]. The disease has caused severe economic losses to the salmon-farming industry in several countries in the northern hemisphere, and confirmed positive diagnostic results for ISA are reportable to the "Office International des Epizooties", OIE [2]. Viruses in the genus *Isavirus *are enveloped particles of 90–140 nm diameter with surface projections consisting of a combined haemagglutinin-esterase (HE) protein [3] and a separate putative fusion (F) protein [4]. The genome is composed of eight segments of linear, single-stranded negative sense RNA ranging in length from 1.0 to 2.4 kb with a total molecular size of approximately 14.3 kb [5]. Sequence analysis of several ISAV isolates on the eight segments consistently reveals two genotypes, one European and one North American. The gene-coding assignments of the ISAV genome differ from those of other orthomyxoviruses [2,4].

Some strains of ISAV can replicate and cause CPE in the CHSE-214 cell line while others do not replicate in this cell line at all [6,7]. The molecular basis for this phenotypic difference is not clearly known. Although there is a strong geographical correlation in that practically all ISAV isolates of the European genotype do not cause CPE in the CHSE cell line, this phenotype is not related to sequence variation in the HE protein [8].

In general, fish viral diseases are difficult to control due to the high susceptibility of fish at an early age, and insufficient knowledge of pathogenesis of virus infections. In this context, we are studying the mechanisms by which ISAV interacts with its host, including the identification of markers for ISAV virulence. Vertebrates [9], including fish [10], mount an early strong innate immune response against virus infections, characterized by the induction and secretion of cytokines, such as type I interferons (IFN-α/β) that mediate an antiviral state. Secreted IFNs signal through a common receptor activating a JAK/STAT signaling pathway which leads to the transcriptional upregulation of numerous IFN-stimulated genes (ISGs), a number of which encode antiviral proteins [10-12]. The induced antiviral proteins include dsRNA-dependent protein kinase R (PKR), 2',5'-oligoadenylate synthetase (OAS), and the Mx proteins [10,12]. Viruses have evolved mechanisms to subvert the host IFN response [12,13]. Previous *in-vitro *studies revealed that ASMx1 inhibited the replication of infectious pancreatic necrosis virus (IPNV), a dsRNA virus belonging to the *Birnaviridae *family, but it did not appear to inhibit replication of ISAV [14,15]. It was concluded from those results that ISAV and IPNV have developed different strategies to avoid the IFN-system of Atlantic salmon. Thus, we were interested in analyzing ISAV strains having phenotypically different growth properties in the CHSE-214 cell line to gain further insight in ISAV virulence. We present evidence that ISAV is sensitive to the antiviral activity of ASMx1.

The following two ISAV isolates were selected for use in this study: NBISA01 and HKS-36. The growth properties of the isolates in CHSE-214 cells have been previously described [6]. NBISA01 is CPE-positive whereas HKS-36 is CPE-negative in CHSE-214 cells. For use, the viruses were propagated and titrated in CHSE-214 cells and/or TO cells as previously described [6,8]. To demonstrate the effects of ASMx1 on the replication of the two ISAV strains, we compared the cytopathogenicity and virus yields of the viruses in normal CHSE-214 cells and in cells constitutively expressing ASMx1. The CHSE-214 cells constitutively expressing ASMx1 [15] were a kind gift from Dr. Børre Robertsen, Norwegian College of Fishery Science, University of Tromsø, Norway. These cells were grown in presence of 0.5 mg/ml zeocine (Invitrogen Life Technologies) to maintain expression of the transfected genes (Dr. Børre Robertsen, personal communication). Virus stocks were prepared from TO cell cultures infected with the selected ISAV isolates which were harvested when CPE was complete; usually 7–9 days post inoculation (dpi). They contained 10^8.16 ^TCID_50_/ml for NBISA01, and 10^7.16^TCID_50_/ml for HKS-36. CHSE-214 cell monolayers in 25 cm^2 ^tissue culture flasks, each inoculated with 1 ml of virus, were monitored daily for CPE and were harvested 14 dpi. Viral titers of the harvests were determined on normal CHSE-214 cells in 48-well tissue culture plates as previously described [6]. A significant delay in development of CPE and a reduction in the severity of CPE, as well as a 10-fold reduction in virus yield were observed for NBISA01 grown in CHSE-214 cells constitutively expressing ASMx1 compared to normal CHSE-214 cells (Table 1). No CPE was seen with HKS-36 in both types of CHSE-214 cells.

**Table 1:** Inhibition of ISAV NBISA01 by AsMx1 in CHSE-214 cells.

CHSE-214 cells^1^	CPE development^2^	CPE severity^3^	Virus titer^4^
Normal	5–14 days	Complete (5+)	5.50
ASMx1 overexpression	6-* days	Incomplete (3+)	4.50

^1^CHSE-214 cells: Normal CHSE-214 cells and CHSE-214 cells constitutively expressing ASMx1.

^2^CPE development denotes time from first appearance to complete CPE in days post inoculation; * indicates incomplete CPE development by 14 days post inoculation.

^3^CPE severity was scored from 1+ to 5+: Complete CPE was scored 5+; Incomplete CPE scored 1+ to 4+).

^4^Virus titers are expressed as TCID_50_/ml.

In an attempt to assess the effects of ASMx1 on the viral mRNA levels, we used real-time RT-PCR in the LightCycler with RNA Amplification Kit SYBR Green I (Roche Applied Science) and PCR primers FA-3/RA-3 targeting a 220-bp product on ISAV segment 8 [7]. This assay has been shown to be 100 times more sensitive than the conventional one-tube RT-PCR assay, and to differentiate ISAV isolates into three CHSE phenotypes (replicating cytopathic, replicating non-cytopathic, and non-replicating) based on their ability to replicate and cause CPE in CHSE-214 cells [7]. Total RNA was extracted from 300 μl of cell culture supernatants of the uninfected and ISAV-infected tissue cultures harvested at 14 dpi by using TRIZOL reagent (Invitrogen Life Technologies). The RNA pellet was dissolved in 10 μl of RNase free water and 1 μl was used in real-time RT-PCR. The assay was performed on three replicates of each virus sample. The thermal conditions were one cycle of reverse transcription at 55°C for 30 min, pre-denaturation at 95°C for 30 s followed by 50 cycles of 95°C for 5s, 59°C for 10s, 72°C for 10s, and data acquisition at 80°C for 2s. Melting curve analysis was performed from 70°C to 95°C in 0.1°C/s increments to assess the specificity of the RT-PCR products. The quantitative (Ct values) and melting curve data were analyzed using LightCycler software version 3.5 (Roche Applied Science). The real-time amplification reaction products were also resolved by 1% agarose gel electrophoresis in 0.5 × TBE buffer and visualized by staining with ethidium bromide and photographed under 304 nm UV light.

**Figure 1:**
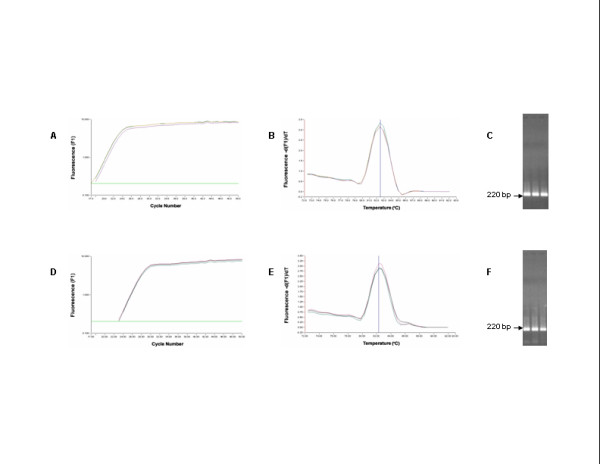
**Amplification, melting curve, and agarose gel electrophoresis of RT-PCR targeting a 220-bp product on ISAV segment 8 using total RNA from ISAV-infected normal CHSE-214 cells: NBISA01 (A-C) and HKS-36 (D-F)**. Total RNA was subjected to real-time RT-PCR with 50 cycle amplification. The arrows in **C **and **F **indicate the expected band of 220 bp on agarose gel.

**Figure 2:**
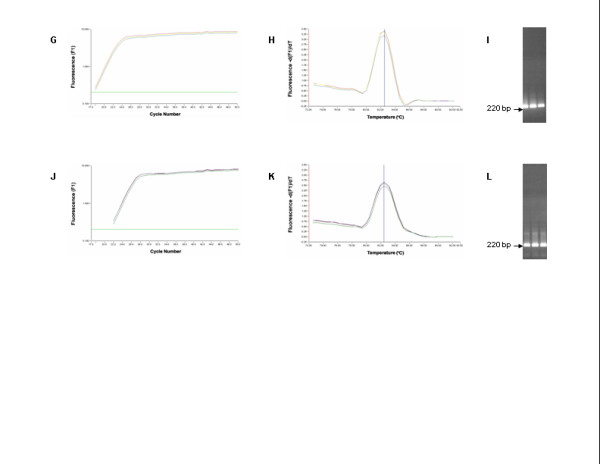
**Amplification, melting curve, and agarose gel electrophoresis of RT-PCR targeting a 220-bp product on ISAV segment 8 using total RNA from ISAV-infected normal CHSE-214 cells constitutively expressing ASMx1: NBISA01 (G-I) and HKS-36 (J-L)**. Total RNA was subjected to real-time RT-PCR with 50 cycle amplification. The arrows in **I **and **L **indicate the expected band of 220 bp on agarose gel.

**Figure 3:**
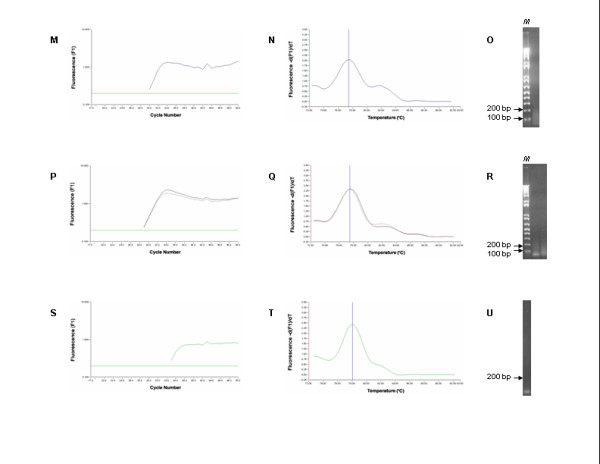
**Amplification, melting curve, and agarose gel electrophoresis of RT-PCR targeting a 220-bp product on ISAV segment 8 using total RNA from uninfected controls: normal CHSE-214 cells (M-O), CHSE-214 cells constitutively expressing ASMx1 (P-R) and the water only control (S-U)**. Samples were subjected to real-time RT-PCR with 50 cycle amplification. The arrows in **O**, **R **and **U **represent DNA markers in lane *M*; note the absence of the 220 bp-band on these agarose gels.

The real-time RT-PCR data are shown in Figures 1,2,3. The mean Ct value of ISAV RNA levels for NBISA01 was 17.39 ± 0.24 in normal CHSE-214 cells (Fig. 1A) and 17.62 ± 0.08 in CHSE-214 cells constitutively expressing ASMx1 (Fig. 2G). For HKS-36, the mean Ct value was 22.94 ± 0.08 in normal CHSE-214 cells (Fig. 1D) and 21.08 ± 0.003 in CHSE-214 cells constitutively expressing ASMx1 (Fig. 2J). For each virus strain, there was no significant difference in the Ct values of both types of CHSE-214 cells. Melting curve analysis showed that the *T*_*m *_of the ISAV templates was 82.5 ± 0.03°C and occurred as a single amplicon peak for both NBISA01 and HKS-36 in normal CHSE-214 cells (Fig. 1B, E) and in CHSE-214 cells constitutively expressing ASMx1 (Fig. 2H, K). All these amplicons had one band of 220 bp on agarose gel (Fig. 1C, F and Fig. 2I, L), indicating virus-specific amplification and uniform virus populations in the individual virus samples. The melting curve analyses of amplification products of uninfected CHSE-214 cells and water control indicated the presence of primer dimers having values of 77.4 ± 0.01°C (Fig. 3N, Q) and 78.1°C (Fig. 3T), respectively. No viral amplicon-specific melting peaks were observed in these samples. The absence of the 220 bp band on agarose gel in these samples (Fig. 3O, R, U) further confirmed the validity of melting curve analyses of these samples. Thus, by real-time RT-PCR we were not able to detect any effect of the ASMx1 activity on ISAV replication for both NBISA01 and HKS-36. In case of NBISA01, this indicated to us that there was no correlation between the virus yields (Table 1) and the Ct values (Fig. 1A and Fig. 2G). This would be expected since the real-time RT-PCR detected both mRNA and viral genomic RNA and was not indicative of infectious virus. Moreover, RNA detected by real-time RT-PCR could be from infectious as well as non-infectious viral progeny. These observations allow us to speculate that the ASMx1 activity on ISAV occurs at the post-transcription steps of virus replication. The human and mouse Mx proteins are known to inhibit different steps of the influenza virus multiplication cycle [16] which is dictated by their intracellular locations. The mouse Mx1 protein accumulates in the nucleus and interferes with primary transcription of influenza virus in the nucleus, whereas the human MxA protein which is localized in the cytoplasm inhibits a subsequent step that presumably takes place in the cytoplasm of infected cells. ASMx1 has been reported to localize in the cytoplasm [15]. Thus in this context, the ASMx1 activity on ISAV may resemble that of the human MxA protein on influenza A virus.

To our knowledge these are the first studies demonstrating that ASMx1 interferes with ISAV replication and suggest that this interference occurs after viral mRNA synthesis. Orthomyxoviruses are known to be sensitive to the antiviral action of type I IFN, and have evolved viral IFN antagonists to suppress IFN induction [17-19]. Influenza A virus NS1 protein binds dsRNA [20] and prevents activation of PKR [21] and OAS [22]. Additionally, the NS1 protein is able to prevent activation of NFκ-B [23] and IRF-3 [24] which are necessary for IFN-β synthesis in virus infected cells. Influenza B virus NS1 protein also inhibits activation of IRF-3 [19], and the activity of ISG15, one of the major type I IFN inducible proteins [25]. Thogoto virus protein ML suppresses IRF-3 function [26]. It is therefore expected that ISAV also encodes protein(s) with IFN antagonistic properties, giving the virus an advantage in its fight against the antiviral host response to infection. The complete ISAV protein profile has not yet been definitively established. ISAV segment 8, like its influenza A virus counterpart, has been shown to encode two proteins; one open reading frame (ORF) was shown to encode a major structural protein corresponding to the virus matrix protein [3], and the other ORF is considered to encode the non-structural protein [2] albeit with no sequence similarity to the influenza NS1 proteins [27]. It would be interesting to see if IFN and ISGs knockdowns would enhance the growth and/or cytopathogenicity of ISAV. This would provide further insight into the exact mechanisms by which ISAV interacts with the Atlantic salmon IFN system with implications for ISAV virulence.

## List of abbreviations

Infectious salmon anaemia virus (ISAV), Atlantic salmon Mx1 protein (ASMx1), cytopathic effect (CPE), Tris borate EDTA (TBE).

## Competing interests

The author(s) declare that they have no competing interests.

## Authors' contributions

MJTK conducted all the experiments and wrote the manuscript. KM assisted with running the real-time RT-PCR assays and with writing the manuscript. FSBK conceived the study, coordinated the research efforts and edited the paper. All three co-authors read and approved the final manuscript.

## References

[B1] Kawaoka Y, Cox NJ, Haller O, Hongo S, Kaverin N, Klenk H-D, Lamb RA, McCauley J, Palese P, Rimstad E, Webster RG, CM Fauquet, MA Mayo, J Maniloff, U Desselberger, LA Bull (2005). Infectious Salmon Anaemia Virus. Virus Taxonomy - Eight Report of the International Comitee on Taxonomy Viruses.

[B2] Kibenge FSB, Munir K, Kibenge MJT, Joseph T, Moneke E (2004). Infectious salmon anaemia virus: causative agent, pathogenesis and immunity. Ani Hlth Res Rev.

[B3] Falk K, Aspehaug V, Vlasak R, Endresen C (2004). Identification andcharacterization of viral structural proteins of infectious salmon anaemia virus. J Virol.

[B4] Aspehaug VT (2005). Characterization of major structural proteins of the infectious salmon anaemia virus (ISAV). Doctoral Thesis.

[B5] Clouthier SC, Rector T, Brown NEC, Anderson ED (2002). Genomic organization of infectious salmon anaemia virus. J Gen Virol.

[B6] Kibenge FSB, Lyaku JR, Rainnie D, Hammell KL (2000). Growth of infectious salmon anaemia virus in CHSE-214 cells and evidence for phenotypic differences between virus strains. J Gen Virol.

[B7] Munir K, Kibenge FSB (2004). Detection of infectious salmon anaemia virus by real-time RT-PCR. J Virol Meth.

[B8] Kibenge FSB, Kibenge MJT, McKenna PK, Stothard P, Marshall R, Cusack RR, McGeachy S (2001). Antigenic variation among isolates of infectious salmon anaemia virus correlates with genetic variation of the viral haemagglutinin gene. J Gen Virol.

[B9] Samuel CE (2001). Antiviral actions of interferons. Clin Microbiol Rev.

[B10] Robertsen B (2006). The interferon system of teleost fish. Fish & Shellfish Immunol.

[B11] Goodbourn S, Didcock L, Randall RE (2000). Interferons: cell signalling, immune modulation, antiviral responses and virus countermeasures. J Gen Virol.

[B12] Stark GR, Kerr IM, Williams BR, Silverman RH, Schreiber RD (1998). How cells respond to interferons. Annu Rev Biochem.

[B13] Garcia-Sastre A (2001). Inhibition of interferon-mediated antiviral responses by influenza A viruses and other negative-strand RNA viruses. Virology.

[B14] Jensen I, Robertsen B (2002). Effect of double-stranded RNA and interferon on the antiviral activity of Atlantic salmon cells against infectious salmon anaemia virus and infectious pancreatic necrosis virus. Fish & Shellfish Immunol.

[B15] Larsen R, Røkenes TP, Robertsen B (2004). Inhibition of infectious pancreatic necrosis virus replication by Atlantic salmon Mx1 protein. J Virol.

[B16] Pavlovic J, Haller O, Staeheli P (1992). Human and mouse Mx proteins inhibit different steps of the influenza virus multiplication cycle. J Virol.

[B17] Garcia-Sastre A, Egorov A, Matassov D, Brandt S, Levy DE (1998). Influenza A virus lacking the NS1 gene replicates in interferon-deficient systems. Virology.

[B18] Hagmaier K, Jennings S, Buse J, Weber F, Kochs G (2003). Novel gene product of *Thogoto virus *segment 6 codes for an interferon antagonist. J Virol.

[B19] Donelan NR, Dauber B, Wang X, Basler CF, Wolff T, Garcia-Sastre A (2004). The N- and C-terminal domains of the NS1 protein of influenza B virus can independently inhibit IRF-3 and beta interferon promoter activation. J Virol.

[B20] Hatada E, Fukuda R (1992). Binding of influenza A virus NS1 protein to dsRNA. J Gen Virol.

[B21] Bergmann M, Garcia-Sastre A, Carnero E, Pehamberger H, Wolff K (2000). Influenza virus NS1 protein counteracts PKR-mediated inhibition of replication. J Virol.

[B22] Lu Y, Wambach M, Katze MG, Krug RM (1995). Binding of the influenza virus NS1 protein to double-stranded RNA inhibits the activation of the protein kinase that phosphorylates the Eif-2 translation initiation factor. Virology.

[B23] Wang X, Li M, Zheng H, Muster T, Palese P (2000). Influenza A virus NS1 protein prevents activation of NF-kappaB and induction of Alpha/Beta interferon. J Virol.

[B24] Talon J, Horvath CM, Polley R, Basler CF, Muster T (2000). Activation of interferon regulatory factor 3 is inhibited by the influenza A virus NS1 protein. J Virol.

[B25] Yuan W, Krug RM (2001). Influenza B virus NS1 protein inhibits conjugation of the interferon (IFN)-induced ubiquitin-like ISG15 protein. EMBO J.

[B26] Jennings S, Martinez-Sobrido L, Garcia-Sastre A, Weber F, Kochs G (2005). Thogoto virus ML protein suppresses IRF3 function. Virology.

[B27] Mjaaland S, Rimstad E, Falk K, Dannevig BH (1997). Genomic characterization of the virus causing infectious salmon anaemia in Atlantic salmon (*Salmo salar L*): an orthomyxo-like virus in a teleost. J Virol.

